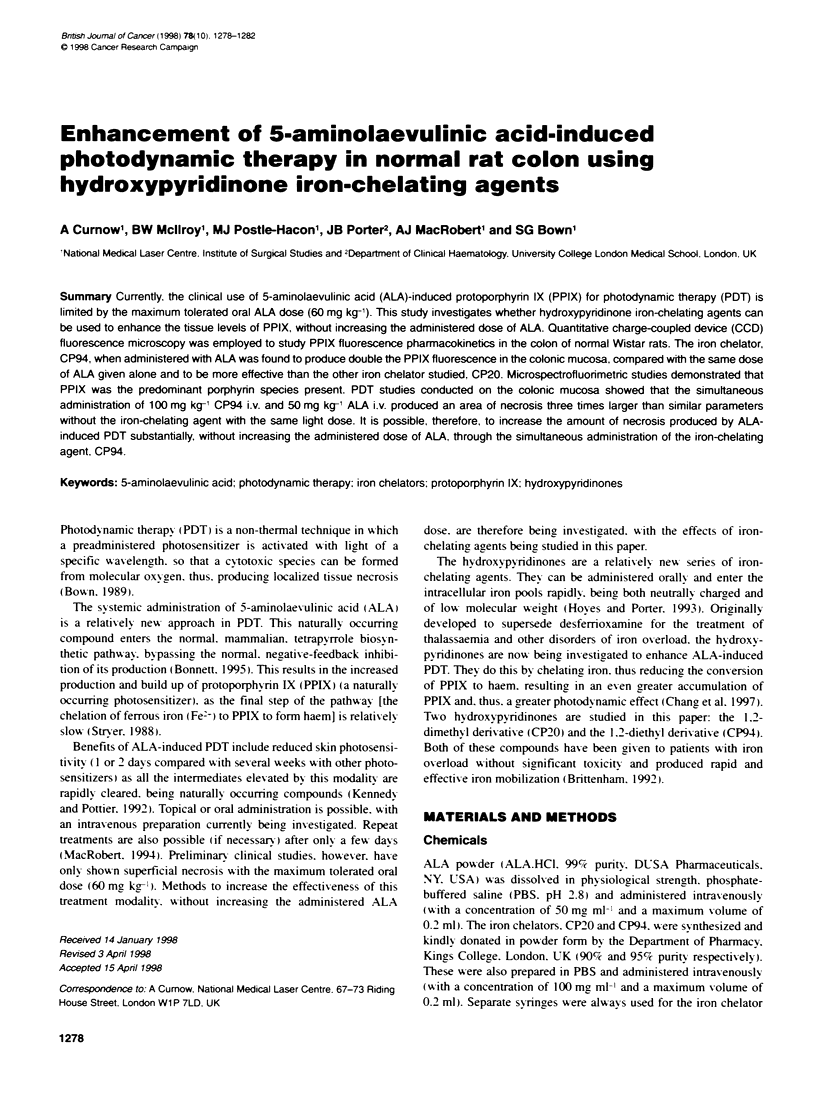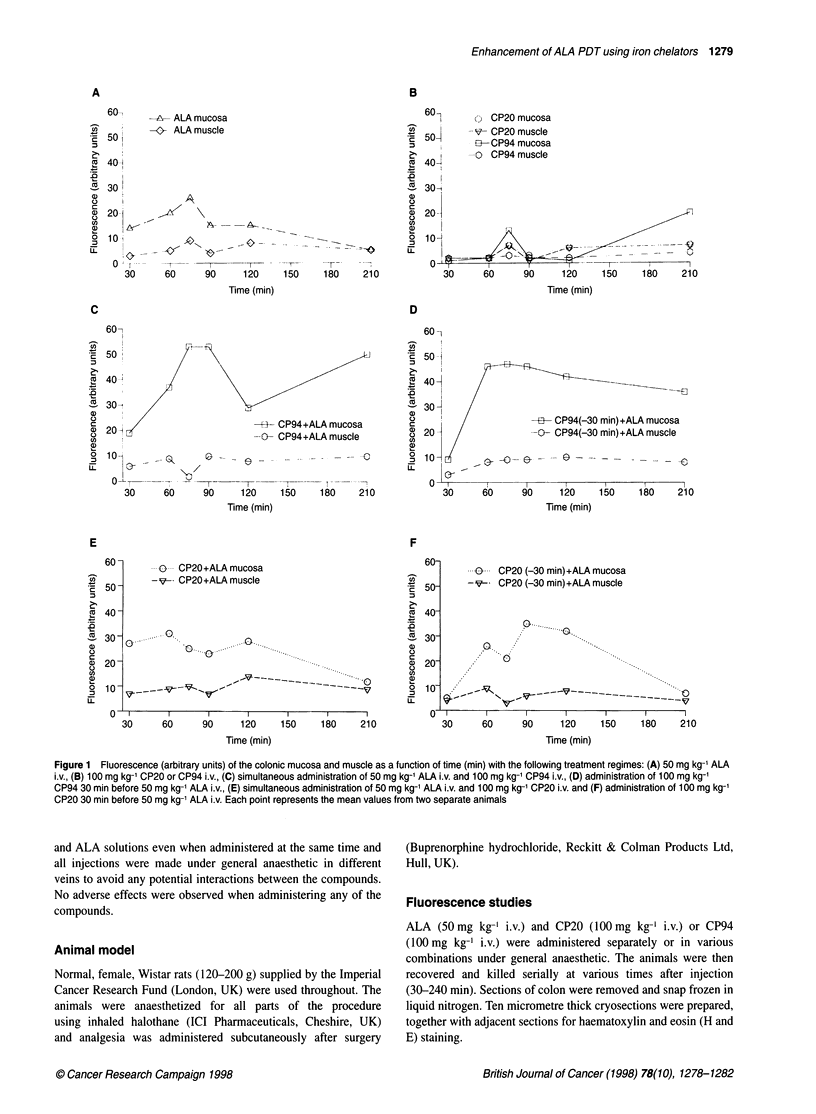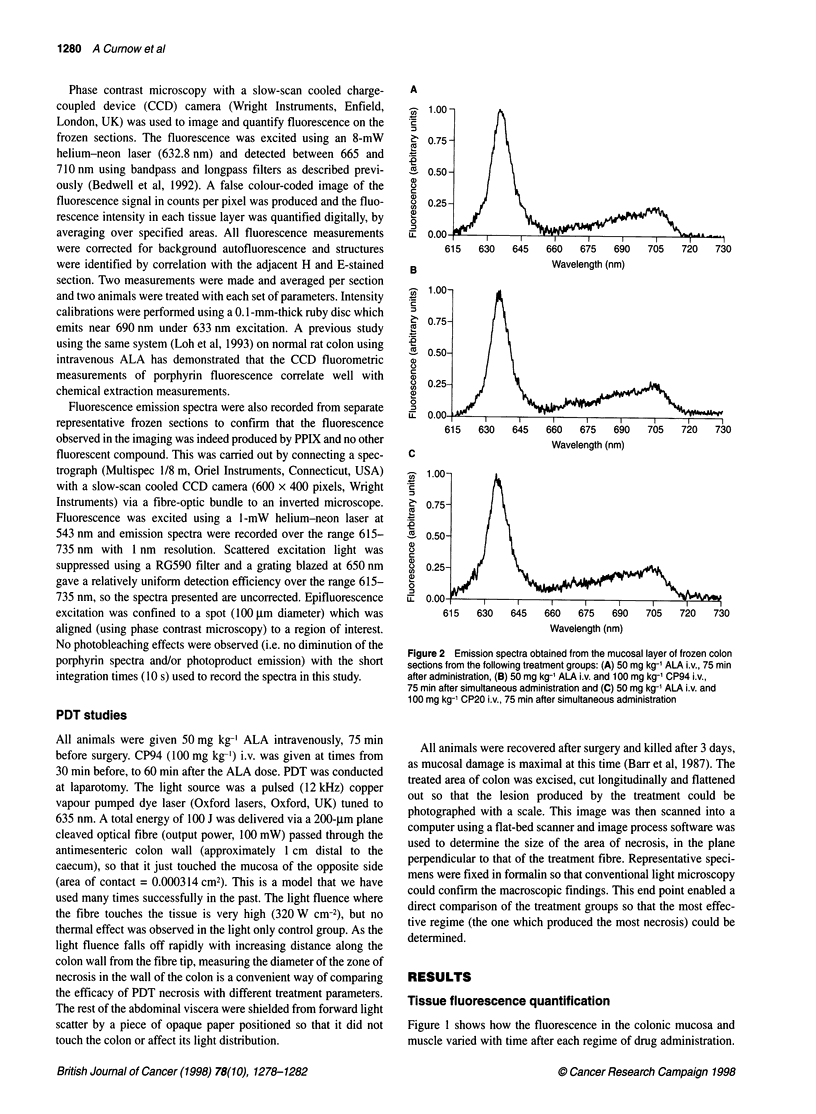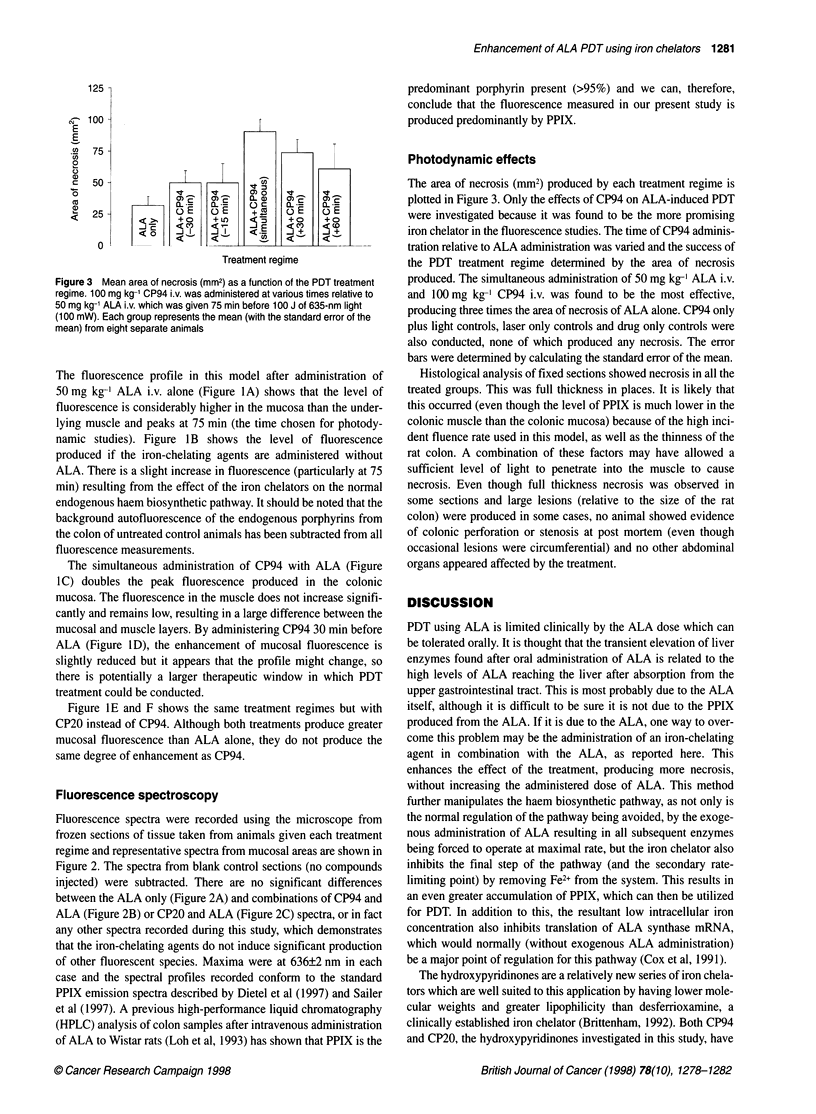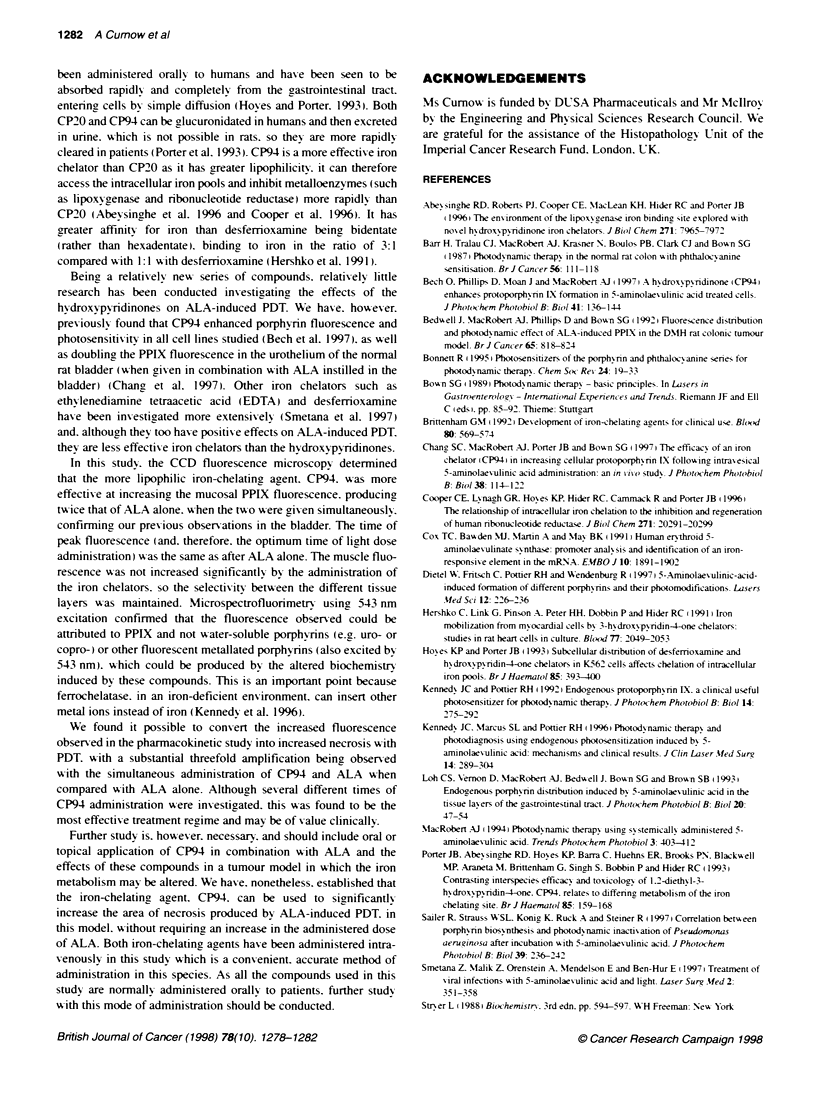# Enhancement of 5-aminolaevulinic acid-induced photodynamic therapy in normal rat colon using hydroxypyridinone iron-chelating agents.

**DOI:** 10.1038/bjc.1998.671

**Published:** 1998-11

**Authors:** A. Curnow, B. W. McIlroy, M. J. Postle-Hacon, J. B. Porter, A. J. MacRobert, S. G. Bown

**Affiliations:** National Medical Laser Centre, Institute of Surgical Studies, University College London Medical School, UK.

## Abstract

Currently, the clinical use of 5-aminolaevulinic acid (ALA)-induced protoporphyrin IX (PPIX) for photodynamic therapy (PDT) is limited by the maximum tolerated oral ALA dose (60 mg kg(-1)). This study investigates whether hydroxypyridinone iron-chelating agents can be used to enhance the tissue levels of PPIX, without increasing the administered dose of ALA. Quantitative charge-coupled device (CCD) fluorescence microscopy was employed to study PPIX fluorescence pharmacokinetics in the colon of normal Wistar rats. The iron chelator, CP94, when administered with ALA was found to produce double the PPIX fluorescence in the colonic mucosa, compared with the same dose of ALA given alone and to be more effective than the other iron chelator studied, CP20. Microspectrofluorimetric studies demonstrated that PPIX was the predominant porphyrin species present. PDT studies conducted on the colonic mucosa showed that the simultaneous administration of 100 mg kg(-1) CP94 i.v. and 50 mg kg(-1) ALA i.v. produced an area of necrosis three times larger than similar parameters without the iron-chelating agent with the same light dose. It is possible, therefore, to increase the amount of necrosis produced by ALA-induced PDT substantially, without increasing the administered dose of ALA, through the simultaneous administration of the iron-chelating agent, CP94.


British Journal of Cancer (1998) 78(10), 1278–1282
© 1998 Cancer Research Campaign

### Enhancement of 5-aminolaevulinic acid-induced photodynamic therapy in normal rat colon using hydroxypyridinone iron-chelating agents

**A Curnow[1], BW McIlroy[1], MJ Postle-Hacon[1], JB Porter[2], AJ MacRobert[1] and SG Bown[1]**

[1]National Medical Laser Centre, Institute of Surgical Studies and [2]Department of Clinical Haematology, University College London Medical School, London, UK

**Summary** Currently, the clinical use of 5-aminolaevulinic acid (ALA)-induced protoporphyrin IX (PPIX) for photodynamic therapy (PDT) is limited by the maximum tolerated oral ALA dose (60 mg $kg^{-1}$). This study investigates whether hydroxypyridinone iron-chelating agents can be used to enhance the tissue levels of PPIX, without increasing the administered dose of ALA. Quantitative charge-coupled device (CCD) fluorescence microscopy was employed to study PPIX fluorescence pharmacokinetics in the colon of normal Wistar rats. The iron chelator, CP94, when administered with ALA was found to produce double the PPIX fluorescence in the colonic mucosa, compared with the same dose of ALA given alone and to be more effective than the other iron chelator studied, CP20. Microspectrofluorimetric studies demonstrated that PPIX was the predominant porphyrin species present. PDT studies conducted on the colonic mucosa showed that the simultaneous administration of 100 mg $kg^{-1}$ CP94 i.v. and 50 mg $kg^{-1}$ ALA i.v. produced an area of necrosis three times larger than similar parameters without the iron-chelating agent with the same light dose. It is possible, therefore, to increase the amount of necrosis produced by ALA-induced PDT substantially, without increasing the administered dose of ALA, through the simultaneous administration of the iron-chelating agent, CP94.

**Keywords:** 5-aminolaevulinic acid; photodynamic therapy; iron chelators; protoporphyrin IX; hydroxypyridinones

Photodynamic therapy (PDT) is a non-thermal technique in which a preadministered photosensitizer is activated with light of a specific wavelength, so that a cytotoxic species can be formed from molecular oxygen, thus, producing localized tissue necrosis (Bown, 1989).

The systemic administration of 5-aminolaevulinic acid (ALA) is a relatively new approach in PDT. This naturally occurring compound enters the normal, mammalian, tetrapyrrole biosynthetic pathway, bypassing the normal, negative-feedback inhibition of its production (Bonnett, 1995). This results in the increased production and build up of protoporphyrin IX (PPIX) (a naturally occurring photosensitizer), as the final step of the pathway [the chelation of ferrous iron ($Fe^{2+}$) to PPIX to form haem] is relatively slow (Stryer, 1988).

Benefits of ALA-induced PDT include reduced skin photosensitivity (1 or 2 days compared with several weeks with other photosensitizers) as all the intermediates elevated by this modality are rapidly cleared, being naturally occurring compounds (Kennedy and Pottier, 1992). Topical or oral administration is possible, with an intravenous preparation currently being investigated. Repeat treatments are also possible (if necessary) after only a few days (MacRobert, 1994). Preliminary clinical studies, however, have only shown superficial necrosis with the maximum tolerated oral dose (60 mg $kg^{-1}$). Methods to increase the effectiveness of this treatment modality, without increasing the administered ALA dose, are therefore being investigated, with the effects of iron-chelating agents being studied in this paper.

The hydroxypyridinones are a relatively new series of iron-chelating agents. They can be administered orally and enter the intracellular iron pools rapidly, being both neutrally charged and of low molecular weight (Hoyes and Porter, 1993). Originally developed to supersede desferrioxamine for the treatment of thalassaemia and other disorders of iron overload, the hydroxypyridinones are now being investigated to enhance ALA-induced PDT. They do this by chelating iron, thus reducing the conversion of PPIX to haem, resulting in an even greater accumulation of PPIX and, thus, a greater photodynamic effect (Chang et al, 1997). Two hydroxypyridinones are studied in this paper: the 1,2-dimethyl derivative (CP20) and the 1,2-diethyl derivative (CP94). Both of these compounds have been given to patients with iron overload without significant toxicity and produced rapid and effective iron mobilization (Brittenham, 1992).

### MATERIALS AND METHODS

### Chemicals

ALA powder (ALA.HCl, 99% purity, DUSA Pharmaceuticals, NY, USA) was dissolved in physiological strength, phosphate-buffered saline (PBS, pH 2.8) and administered intravenously (with a concentration of 50 mg $ml^{-1}$ and a maximum volume of 0.2 ml). The iron chelators, CP20 and CP94, were synthesized and kindly donated in powder form by the Department of Pharmacy, Kings College, London, UK (90% and 95% purity respectively). These were also prepared in PBS and administered intravenously (with a concentration of 100 mg $ml^{-1}$ and a maximum volume of 0.2 ml). Separate syringes were always used for the iron chelator

*Received 14 January 1998*

*Revised 3 April 1998*

*Accepted 15 April 1998*

*Correspondence to:* A Curnow, National Medical Laser Centre, 67–73 Riding House Street, London W1P 7LD, UK

**Figure 1** Fluorescence (arbitrary units) of the colonic mucosa and muscle as a function of time (min) with the following treatment regimes: (**A**) 50 mg $kg^{-1}$ ALA i.v., (**B**) 100 mg $kg^{-1}$ CP20 or CP94 i.v., (**C**) simultaneous administration of 50 mg $kg^{-1}$ ALA i.v. and 100 mg $kg^{-1}$ CP94 i.v., (**D**) administration of 100 mg $kg^{-1}$ CP94 30 min before 50 mg $kg^{-1}$ ALA i.v., (**E**) simultaneous administration of 50 mg $kg^{-1}$ ALA i.v. and 100 mg $kg^{-1}$ CP20 i.v. and (**F**) administration of 100 mg $kg^{-1}$ CP20 30 min before 50 mg $kg^{-1}$ ALA i.v. Each point represents the mean values from two separate animals and ALA solutions even when administered at the same time and all injections were made under general anaesthetic in different veins to avoid any potential interactions between the compounds. No adverse effects were observed when administering any of the compounds.

### Animal model

Normal, female, Wistar rats (120–200 g) supplied by the Imperial Cancer Research Fund (London, UK) were used throughout. The animals were anaesthetized for all parts of the procedure using inhaled halothane (ICI Pharmaceuticals, Cheshire, UK) and analgesia was administered subcutaneously after surgery (Buprenorphine hydrochloride, Reckitt & Colman Products Ltd, Hull, UK).

### Fluorescence studies

ALA (50 mg $kg^{-1}$ i.v.) and CP20 (100 mg $kg^{-1}$ i.v.) or CP94 (100 mg $kg^{-1}$ i.v.) were administered separately or in various combinations under general anaesthetic. The animals were then recovered and killed serially at various times after injection (30–240 min). Sections of colon were removed and snap frozen in liquid nitrogen. Ten micrometre thick cryosections were prepared, together with adjacent sections for haematoxylin and eosin (H and E) staining.

© Cancer Research Campaign 1998

Phase contrast microscopy with a slow-scan cooled charge-coupled device (CCD) camera (Wright Instruments, Enfield, London, UK) was used to image and quantify fluorescence on the frozen sections. The fluorescence was excited using an 8-mW helium–neon laser (632.8 nm) and detected between 665 and 710 nm using bandpass and longpass filters as described previously (Bedwell et al, 1992). A false colour-coded image of the fluorescence signal in counts per pixel was produced and the fluorescence intensity in each tissue layer was quantified digitally, by averaging over specified areas. All fluorescence measurements were corrected for background autofluorescence and structures were identified by correlation with the adjacent H and E-stained section. Two measurements were made and averaged per section and two animals were treated with each set of parameters. Intensity calibrations were performed using a 0.1-mm-thick ruby disc which emits near 690 nm under 633 nm excitation. A previous study using the same system (Loh et al, 1993) on normal rat colon using intravenous ALA has demonstrated that the CCD fluorometric measurements of porphyrin fluorescence correlate well with chemical extraction measurements.

Fluorescence emission spectra were also recorded from separate representative frozen sections to confirm that the fluorescence observed in the imaging was indeed produced by PPIX and no other fluorescent compound. This was carried out by connecting a spectrograph (Multispec 1/8 m, Oriel Instruments, Connecticut, USA) with a slow-scan cooled CCD camera (600 × 400 pixels, Wright Instruments) via a fibre-optic bundle to an inverted microscope. Fluorescence was excited using a 1-mW helium–neon laser at 543 nm and emission spectra were recorded over the range 615–735 nm with 1 nm resolution. Scattered excitation light was suppressed using a RG590 filter and a grating blazed at 650 nm gave a relatively uniform detection efficiency over the range 615–735 nm, so the spectra presented are uncorrected. Epifluorescence excitation was confined to a spot (100 μm diameter) which was aligned (using phase contrast microscopy) to a region of interest. No photobleaching effects were observed (i.e. no diminution of the porphyrin spectra and/or photoproduct emission) with the short integration times (10 s) used to record the spectra in this study.

**Figure 2** Emission spectra obtained from the mucosal layer of frozen colon sections from the following treatment groups: (**A**) 50 mg $kg^{-1}$ ALA i.v., 75 min after administration, (**B**) 50 mg $kg^{-1}$ ALA i.v. and 100 mg $kg^{-1}$ CP94 i.v., 75 min after simultaneous administration and (**C**) 50 mg $kg^{-1}$ ALA i.v. and 100 mg $kg^{-1}$ CP20 i.v., 75 min after simultaneous administration

### PDT studies

All animals were given 50 mg $kg^{-1}$ ALA intravenously, 75 min before surgery. CP94 (100 mg $kg^{-1}$) i.v. was given at times from 30 min before, to 60 min after the ALA dose. PDT was conducted at laparotomy. The light source was a pulsed (12 kHz) copper vapour pumped dye laser (Oxford lasers, Oxford, UK) tuned to 635 nm. A total energy of 100 J was delivered via a 200-μm plane cleaved optical fibre (output power, 100 mW) passed through the antimesenteric colon wall (approximately 1 cm distal to the caecum), so that it just touched the mucosa of the opposite side (area of contact = 0.000314 $cm^2$). This is a model that we have used many times successfully in the past. The light fluence where the fibre touches the tissue is very high (320 W $cm^{-2}$), but no thermal effect was observed in the light only control group. As the light fluence falls off rapidly with increasing distance along the colon wall from the fibre tip, measuring the diameter of the zone of necrosis in the wall of the colon is a convenient way of comparing the efficacy of PDT necrosis with different treatment parameters. The rest of the abdominal viscera were shielded from forward light scatter by a piece of opaque paper positioned so that it did not touch the colon or affect its light distribution.

All animals were recovered after surgery and killed after 3 days, as mucosal damage is maximal at this time (Barr et al, 1987). The treated area of colon was excised, cut longitudinally and flattened out so that the lesion produced by the treatment could be photographed with a scale. This image was then scanned into a computer using a flat-bed scanner and image process software was used to determine the size of the area of necrosis, in the plane perpendicular to that of the treatment fibre. Representative specimens were fixed in formalin so that conventional light microscopy could confirm the macroscopic findings. This end point enabled a direct comparison of the treatment groups so that the most effective regime (the one which produced the most necrosis) could be determined.

### RESULTS

### Tissue fluorescence quantification

Figure 1 shows how the fluorescence in the colonic mucosa and muscle varied with time after each regime of drug administration.

© Cancer Research Campaign 1998

**Figure 3** Mean area of necrosis ($mm^2$) as a function of the PDT treatment regime. 100 mg $kg^{-1}$ CP94 i.v. was administered at various times relative to 50 mg $kg^{-1}$ ALA i.v. which was given 75 min before 100 J of 635-nm light (100 mW). Each group represents the mean (with the standard error of the mean) from eight separate animals

The fluorescence profile in this model after administration of 50 mg $kg^{-1}$ ALA i.v. alone (Figure 1A) shows that the level of fluorescence is considerably higher in the mucosa than the underlying muscle and peaks at 75 min (the time chosen for photodynamic studies). Figure 1B shows the level of fluorescence produced if the iron-chelating agents are administered without ALA. There is a slight increase in fluorescence (particularly at 75 min) resulting from the effect of the iron chelators on the normal endogenous haem biosynthetic pathway. It should be noted that the background autofluorescence of the endogenous porphyrins from the colon of untreated control animals has been subtracted from all fluorescence measurements.

The simultaneous administration of CP94 with ALA (Figure 1C) doubles the peak fluorescence produced in the colonic mucosa. The fluorescence in the muscle does not increase significantly and remains low, resulting in a large difference between the mucosal and muscle layers. By administering CP94 30 min before ALA (Figure 1D), the enhancement of mucosal fluorescence is slightly reduced but it appears that the profile might change, so there is potentially a larger therapeutic window in which PDT treatment could be conducted.

Figure 1E and F shows the same treatment regimes but with CP20 instead of CP94. Although both treatments produce greater mucosal fluorescence than ALA alone, they do not produce the same degree of enhancement as CP94.

### Fluorescence spectroscopy

Fluorescence spectra were recorded using the microscope from frozen sections of tissue taken from animals given each treatment regime and representative spectra from mucosal areas are shown in Figure 2. The spectra from blank control sections (no compounds injected) were subtracted. There are no significant differences between the ALA only (Figure 2A) and combinations of CP94 and ALA (Figure 2B) or CP20 and ALA (Figure 2C) spectra, or in fact any other spectra recorded during this study, which demonstrates that the iron-chelating agents do not induce significant production of other fluorescent species. Maxima were at 636±2 nm in each case and the spectral profiles recorded conform to the standard PPIX emission spectra described by Dietel et al (1997) and Sailer et al (1997). A previous high-performance liquid chromatography (HPLC) analysis of colon samples after intravenous administration of ALA to Wistar rats (Loh et al, 1993) has shown that PPIX is the predominant porphyrin present (>95%) and we can, therefore, conclude that the fluorescence measured in our present study is produced predominantly by PPIX.

### Photodynamic effects

The area of necrosis ($mm^2$) produced by each treatment regime is plotted in Figure 3. Only the effects of CP94 on ALA-induced PDT were investigated because it was found to be the more promising iron chelator in the fluorescence studies. The time of CP94 administration relative to ALA administration was varied and the success of the PDT treatment regime determined by the area of necrosis produced. The simultaneous administration of 50 mg $kg^{-1}$ ALA i.v. and 100 mg $kg^{-1}$ CP94 i.v. was found to be the most effective, producing three times the area of necrosis of ALA alone. CP94 only plus light controls, laser only controls and drug only controls were also conducted, none of which produced any necrosis. The error bars were determined by calculating the standard error of the mean.

Histological analysis of fixed sections showed necrosis in all the treated groups. This was full thickness in places. It is likely that this occurred (even though the level of PPIX is much lower in the colonic muscle than the colonic mucosa) because of the high incident fluence rate used in this model, as well as the thinness of the rat colon. A combination of these factors may have allowed a sufficient level of light to penetrate into the muscle to cause necrosis. Even though full thickness necrosis was observed in some sections and large lesions (relative to the size of the rat colon) were produced in some cases, no animal showed evidence of colonic perforation or stenosis at post mortem (even though occasional lesions were circumferential) and no other abdominal organs appeared affected by the treatment.

### DISCUSSION

PDT using ALA is limited clinically by the ALA dose which can be tolerated orally. It is thought that the transient elevation of liver enzymes found after oral administration of ALA is related to the high levels of ALA reaching the liver after absorption from the upper gastrointestinal tract. This is most probably due to the ALA itself, although it is difficult to be sure it is not due to the PPIX produced from the ALA. If it is due to the ALA, one way to overcome this problem may be the administration of an iron-chelating agent in combination with the ALA, as reported here. This enhances the effect of the treatment, producing more necrosis, without increasing the administered dose of ALA. This method further manipulates the haem biosynthetic pathway, as not only is the normal regulation of the pathway being avoided, by the exogenous administration of ALA resulting in all subsequent enzymes being forced to operate at maximal rate, but the iron chelator also inhibits the final step of the pathway (and the secondary rate-limiting point) by removing $Fe^{2+}$ from the system. This results in an even greater accumulation of PPIX, which can then be utilized for PDT. In addition to this, the resultant low intracellular iron concentration also inhibits translation of ALA synthase mRNA, which would normally (without exogenous ALA administration) be a major point of regulation for this pathway (Cox et al, 1991).

The hydroxypyridinones are a relatively new series of iron chelators which are well suited to this application by having lower molecular weights and greater lipophilicity than desferrioxamine, a clinically established iron chelator (Brittenham, 1992). Both CP94 and CP20, the hydroxypyridinones investigated in this study, have been administered orally to humans and have been seen to be absorbed rapidly and completely from the gastrointestinal tract, entering cells by simple diffusion (Hoyes and Porter, 1993). Both CP20 and CP94 can be glucuronidated in humans and then excreted in urine, which is not possible in rats, so they are more rapidly cleared in patients (Porter et al, 1993). CP94 is a more effective iron chelator than CP20 as it has greater lipophilicity, it can therefore access the intracellular iron pools and inhibit metalloenzymes (such as lipoxygenase and ribonucleotide reductase) more rapidly than CP20 (Abeysinghe et al, 1996 and Cooper et al, 1996). It has greater affinity for iron than desferrioxamine being bidentate (rather than hexadentate), binding to iron in the ratio of 3:1 compared with 1:1 with desferrioxamine (Hershko et al, 1991).

Being a relatively new series of compounds, relatively little research has been conducted investigating the effects of the hydroxypyridinones on ALA-induced PDT. We have, however, previously found that CP94 enhanced porphyrin fluorescence and photosensitivity in all cell lines studied (Bech et al, 1997), as well as doubling the PPIX fluorescence in the urothelium of the normal rat bladder (when given in combination with ALA instilled in the bladder) (Chang et al, 1997). Other iron chelators such as ethylenediamine tetraacetic acid (EDTA) and desferrioxamine have been investigated more extensively (Smetana et al, 1997) and, although they too have positive effects on ALA-induced PDT, they are less effective iron chelators than the hydroxypyridinones.

In this study, the CCD fluorescence microscopy determined that the more lipophilic iron-chelating agent, CP94, was more effective at increasing the mucosal PPIX fluorescence, producing twice that of ALA alone, when the two were given simultaneously, confirming our previous observations in the bladder. The time of peak fluorescence (and, therefore, the optimum time of light dose administration) was the same as after ALA alone. The muscle fluorescence was not increased significantly by the administration of the iron chelators, so the selectivity between the different tissue layers was maintained. Microspectrofluorimetry using 543 nm excitation confirmed that the fluorescence observed could be attributed to PPIX and not water-soluble porphyrins (e.g. uro- or copro-) or other fluorescent metallated porphyrins (also excited by 543 nm), which could be produced by the altered biochemistry induced by these compounds. This is an important point because ferrochelatase, in an iron-deficient environment, can insert other metal ions instead of iron (Kennedy et al, 1996).

We found it possible to convert the increased fluorescence observed in the pharmacokinetic study into increased necrosis with PDT, with a substantial threefold amplification being observed with the simultaneous administration of CP94 and ALA when compared with ALA alone. Although several different times of CP94 administration were investigated, this was found to be the most effective treatment regime and may be of value clinically.

Further study is, however, necessary, and should include oral or topical application of CP94 in combination with ALA and the effects of these compounds in a tumour model in which the iron metabolism may be altered. We have, nonetheless, established that the iron-chelating agent, CP94, can be used to significantly increase the area of necrosis produced by ALA-induced PDT, in this model, without requiring an increase in the administered dose of ALA. Both iron-chelating agents have been administered intravenously in this study which is a convenient, accurate method of administration in this species. As all the compounds used in this study are normally administered orally to patients, further study with this mode of administration should be conducted.

### ACKNOWLEDGEMENTS

Ms Curnow is funded by DUSA Pharmaceuticals and Mr McIlroy by the Engineering and Physical Sciences Research Council. We are grateful for the assistance of the Histopathology Unit of the Imperial Cancer Research Fund, London, UK.

### REFERENCES

Abeysinghe RD, Roberts PJ, Cooper CE, MacLean KH, Hider RC and Porter JB (1996) The environment of the lipoxygenase iron binding site explored with novel hydroxypyridinone iron chelators. *J Biol Chem* **271**: 7965–7972

Barr H, Tralau CJ, MacRobert AJ, Krasner N, Boulos PB, Clark CJ and Bown SG (1987) Photodynamic therapy in the normal rat colon with phthalocyanine sensitisation. *Br J Cancer* **56**: 111–118

Bech O, Phillips D, Moan J and MacRobert AJ (1997) A hydroxypyridinone (CP94) enhances protoporphyrin IX formation in 5-aminolaevulinic acid treated cells. *J Photochem Photobiol B: Biol* **41**: 136–144

Bedwell J, MacRobert AJ, Phillips D and Bown SG (1992) Fluorescence distribution and photodynamic effect of ALA-induced PPIX in the DMH rat colonic tumour model. *Br J Cancer* **65**: 818–824

Bonnett R (1995) Photosensitizers of the porphyrin and phthalocyanine series for photodynamic therapy. *Chem Soc Rev* **24**: 19–33

Bown SG (1989) Photodynamic therapy – basic principles. In *Lasers in Gastroenterology – International Experiences and Trends*. Riemann JF and Ell C (eds), pp. 85–92. Thieme: Stuttgart

Brittenham GM (1992) Development of iron-chelating agents for clinical use. *Blood* **80**: 569–574

Chang SC, MacRobert AJ, Porter JB and Bown SG (1997) The efficacy of an iron chelator (CP94) in increasing cellular protoporphyrin IX following intravesical 5-aminolaevulinic acid administration: an *in vivo* study. *J Photochem Photobiol B: Biol* **38**: 114–122

Cooper CE, Lynagh GR, Hoyes KP, Hider RC, Cammack R and Porter JB (1996) The relationship of intracellular iron chelation to the inhibition and regeneration of human ribonucleotide reductase. *J Biol Chem* **271**: 20291–20299

Cox TC, Bawden MJ, Martin A and May BK (1991) Human erythroid 5-aminolaevulinate synthase: promoter analysis and identification of an iron-responsive element in the mRNA. *EMBO J* **10**: 1891–1902

Dietel W, Fritsch C, Pottier RH and Wendenburg R (1997) 5-Aminolaevulinic-acid-induced formation of different porphyrins and their photomodifications. *Lasers Med Sci* **12**: 226–236

Hershko C, Link G, Pinson A, Peter HH, Dobbin P and Hider RC (1991) Iron mobilization from myocardial cells by 3-hydroxypyridin-4-one chelators: studies in rat heart cells in culture. *Blood* **77**: 2049–2053

Hoyes KP and Porter JB (1993) Subcellular distribution of desferrioxamine and hydroxypyridin-4-one chelators in K562 cells affects chelation of intracellular iron pools. *Br J Haematol* **85**: 393–400

Kennedy JC and Pottier RH (1992) Endogenous protoporphyrin IX, a clinical useful photosensitizer for photodynamic therapy. *J Photochem Photobiol B: Biol* **14**: 275–292

Kennedy JC, Marcus SL and Pottier RH (1996) Photodynamic therapy and photodiagnosis using endogenous photosensitization induced by 5-aminolaevulinic acid: mechanisms and clinical results. *J Clin Laser Med Surg* **14**: 289–304

Loh CS, Vernon D, MacRobert AJ, Bedwell J, Bown SG and Brown SB (1993) Endogenous porphyrin distribution induced by 5-aminolaevulinic acid in the tissue layers of the gastrointestinal tract. *J Photochem Photobiol B: Biol* **20**: 47–54

MacRobert AJ (1994) Photodynamic therapy using systemically administered 5-aminolaevulinic acid. *Trends Photochem Photobiol* **3**: 403–412

Porter JB, Abeysinghe RD, Hoyes KP, Barra C, Huehns ER, Brooks PN, Blackwell MP, Araneta M, Brittenham G, Singh S, Bobbin P and Hider RC (1993) Contrasting interspecies efficacy and toxicology of 1,2-diethyl-3-hydroxypyridin-4-one, CP94, relates to differing metabolism of the iron chelating site. *Br J Haematol* **85**: 159–168

Sailer R, Strauss WSL, Konig K, Ruck A and Steiner R (1997) Correlation between porphyrin biosynthesis and photodynamic inactivation of *Pseudomonas aeruginosa* after incubation with 5-aminolaevulinic acid. *J Photochem Photobiol B: Biol* **39**: 236–242

Smetana Z, Malik Z, Orenstein A, Mendelson E and Ben-Hur E (1997) Treatment of viral infections with 5-aminolaevulinic acid and light. *Laser Surg Med* **2**: 351–358

Stryer L (1988) *Biochemistry*, 3rd edn, pp. 594–597. WH Freeman: New York

© Cancer Research Campaign 1998